# Differences in Marine Toxin Poisonings Reported to US Poison Centers After Pandemic Restrictions

**DOI:** 10.3390/toxins17090444

**Published:** 2025-09-04

**Authors:** Baylin J. Bennett, Cailee Hill, Hugh B. Roland, Lorraine C. Backer, Amy H. Schnall, Matthew O. Gribble

**Affiliations:** 1 Department of Medicine, Division of Occupational, Environmental and Climate Medicine, University of California, 490 Illinois Street, San Francisco, CA 94158, USA; matthew.gribble@ucsf.edu; 2 Oak Ridge Institute for Science and Education, Oak Ridge, TN 37830, USA; ssx2@cdc.gov; 3 National Center for Environmental Health, Centers for Disease Control and Prevention, Atlanta, GA 30341, USA; lfb9@cdc.gov (L.C.B.); ghu5@cdc.gov (A.H.S.); 4 Department of Health Policy and Organization, School of Public Health, University of Alabama at Birmingham, Birmingham, AL 35294, USA; hbroland@uab.edu

**Keywords:** marine toxins, seafood, harmful algal blooms, epidemiology, surveillance, OneHealth

## Abstract

This study investigated whether marine toxin poisonings reported to U.S. Poison Centers changed during the height of the pandemic period (April 2020 to December 2021). The National Poison Data System was queried for single-substance human exposure calls between 1 January 2000 and 31 March 2022 pertaining to ichthyosarcotoxins. Incidence rate ratios for exposure calls were calculated using mixed-effects negative binomial regression. Call counts were aggregated by year and regressed on a binary indicator for occurrence during pandemic restrictions. During the peak pandemic period, exposure calls decreased for several toxins: ciguatera poisoning: 0.57 (0.43, 0.76); clupeotoxic fish poisoning: 0.12 (0.04, 0.39); diarrhetic shellfish poisoning: 0.28 (0.16, 0.49); paralytic shellfish poisoning: 0.23 (0.17, 0.33); scombroid fish poisoning: 0.46 (0.36, 0.57). However, palytoxin poisoning (1.94 (1.32, 2.84)) and tetrodotoxin poisoning (1.73 (1.46, 2.04)) exposure calls appear to have increased. All results were Bonferroni-significant (*p* ≤ 0.0009). Sensitivity analyses suggest the PLTX increase began prior to pandemic restrictions, whereas the TTX increase appeared to be directly associated with the restrictions. Both men and women reported increases in TTX exposure calls. The TTX increase could be associated with potentially increased participation in outdoor activities, as TTX exposures are linked to amphibia, echinoderms, fish, and mollusks, among other animals.

## 1. Introduction

As the halfway point of the United Nations Ocean Decade for Sustainable Development (2021–2030) approaches [1], it is timely to explore and clarify patterns of exposure reports related to human interactions with marine life [2]. In this study, we investigate how marine toxin exposures reported to U.S. poison centers (PCs) may have changed during the COVID-19 pandemic era restrictions on travel and business activities, implemented from April 2020 to December 2021 in the United States. We use this period of restrictions as a proxy for behavioral changes to understand how dramatic shifts in behaviors may influence exposures to different marine toxins. Scholars have made the case for the evaluation of marine toxin exposure risks in diverse settings and circumstances to elucidate factors influencing risk in different contexts globally [3]. Investigating trends in reporting of marine toxin exposures during this social shock, including trends by poisoning type and sex, may be revealing of behavioral changes important to our understanding of both exposure risk and risk reduction.

## 2. Results

Results are presented in Table 1. During pandemic restrictions, PLTX and TTX exposure calls increased by 94% (incidence rate ratio [IRR]: 1.94; confidence intervals [CI]: 1.32, 2.84) and 73% (IRR: 1.73; CI: 1.46, 2.04), respectively. Reported exposure calls for CFP, CLP, DSP, PSP, and SFP decreased. Except for AZP and DAP, all results were Bonferroni-significant (i.e., *p* ≤ 0.0009). Sex-stratified results showed a 112% increase in PLTX exposure calls from men (IRR: 2.12; CI: 1.47, 3.07), while the increase in calls from women was not statistically significant (IRR: 1.87; CI: 0.97, 3.58). Sex-stratified results showed increases in TTX exposure calls from both men (IRR: 1.83; CI: 1.51, 2.22) and women (IRR: 1.73; CI: 1.38, 2.16). Sex-stratified results for the other evaluated toxins showed decreasing calls, although not all changes were significant.

For the toxins that increased during the pandemic restriction period (i.e., PLTX and TTX), the sensitivity analyses showed that TTX reports increased (IRR: 1.71; CI: 1.37, 2.15) (see Table 2) but PLTX reports did not increase significantly (IRR: 0.93; CI: 0.67, 1.28). When stratified by sex, TTX reports from women (IRR: 1.69; CI: 1.29, 2.23) and men (IRR: 1.86; CI: 1.45, 2.38) both increased.

**Figure 1:**
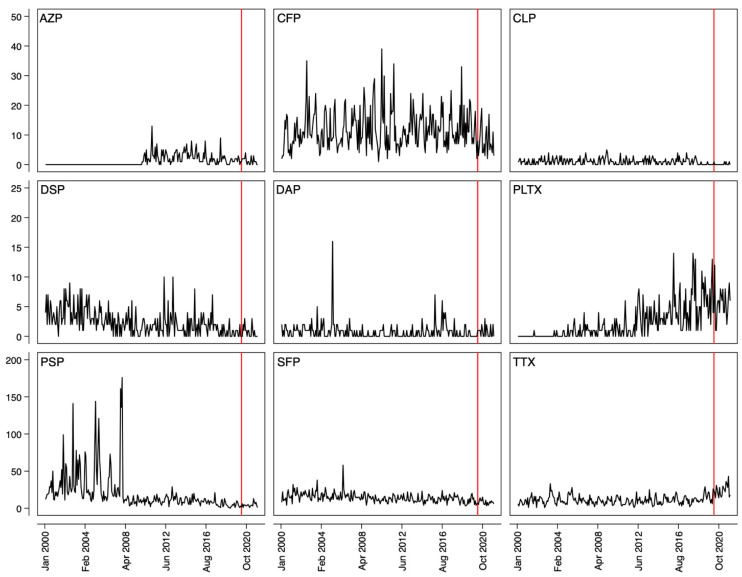
Calls to the Poison Centers reporting different marine toxin poisonings in US states and territories between January 2000 and December 2021. The red line marks April 2020, which was the start of pandemic restriction period as defined in our study [4]. AZP = azaspiracid shellfish poisoning; CFP = ciguatera fish poisoning; CLP = clupeotoxic poisoning; DSP = diarrhetic shellfish poisoning; DAP = domoic acid poisoning; PLTX = palytoxin poisoning; PSP = paralytic shellfish poisoning; SFP = scombroid fish poisoning; TTX = tetrodotoxin poisoning.

Figure 1 shows call counts for the nine ichthyosarcotoxins. PLTX trends may have preceded the pandemic.

## 3. Discussion

This study finds that most calls to PCs regarding marine toxin exposures decreased following pandemic restrictions; however, reports of PLTX and TTX exposures increased. Further, the apparent increase in PLTX reports was nullified by sensitivity analyses, whereas the increase in TTX reports was strengthened.

Decreased reports of most marine toxin exposure calls during pandemic restrictions may relate to decreased access to and consumption of origin species due to restaurant closures and decreased importation of fish because exposures are typically from seafood. However, TTX report increases require further scrutiny. One possible explanation is greater participation in outdoor activities during pandemic restrictions, such as subsistence harvesting and outdoor recreation, as TTX is linked to amphibia, echinoderms, fish, and mollusks, among other animals [5]. Additionally, evidence suggests a potential interaction exists between COVID-19 infection and marine toxin exposure [6,7].

We note several limitations to this study. The NPDS relies on data reported to PCs voluntarily by healthcare providers and the public; therefore, the NPDS does not capture all exposures, and the true denominator of exposed people is unknown. Our outcomes are not confirmed cases, so we cannot be sure the toxin was responsible for the health effects without further clinical or laboratory confirmation. Additionally, due to the limited sample size we could not stratify the data by potential effect modifiers such as age and symptom severity. Further, the state or territory coded is based on the location of the initial call, not necessarily where the exposure occurred, and individual exposure details (e.g., exposure source) were also limited.

Future studies may assess whether trends in toxin reports have returned to pre-restriction period levels or whether they are still reduced. Research might also investigate possible associations between decreased reported exposures and changes in industries directly tied to toxins (e.g., the seafood industry) and in harvesting behaviors. State- and county-level scrutiny may investigate regional heterogeneity and provide insight into possible sources of difference, such as coastal populations possibly being less impacted by pandemic restrictions as they relate to toxin exposures due to their proximity to potential exposure sources.

## 4. Conclusions

In assessing shifts in reported exposures corresponding with the COVID-19 pandemic restrictions and related behavior changes, this study seeks to better understand how behavioral changes may influence exposure risk to various marine toxins. While the increase in PLTX reports during pandemic restrictions was not significant, recent market data have reported an expected increase in the aquaria trade [8], and PLTX exposures are linked to personal and commercial aquaria activities. Marine toxins, including algal toxins and ichthyosarcotoxins, burden communities with impacts that are felt environmentally (e.g., food web disruption through fish kills and habitat loss [9]), medically (e.g., encumbering both human and wildlife medicine [10,11]), and economically (e.g., reductions in tourism, recreation, and commercial fisheries activities [12]). Harmful algal blooms may be increasing in frequency, magnitude, and unpredictability [13], and toxins may shift geographic distribution [14]. Societal responses to a changing toxin environment (e.g., dietary behaviors) may influence subsequent poisoning risks.

## 5. Materials and Methods

The National Poison Data System (NPDS) is a surveillance system owned and operated by America’s Poison Centers that collects call data and exposure information from all PCs in the United States. NPDS data are uploaded in near-real-time, approximately every five minutes. We queried the NPDS for calls between 1 January 2000 and 31 March 2022 to U.S. PCs coded as single-substance human exposures to one of the generic marine toxins under the NPDS category ichthyosarcotoxins. We excluded calls in which the medical outcome was coded as “unrelated effect,” “the exposure was probably not responsible for the effect(s),”or “confirmed non-exposure,” as well as calls coded with multiple marine toxins (e.g., ciguatera and scombroid) or a single marine toxin and another substance (e.g., ciguatera and antihistamines). We also excluded calls (*n* = 193) where the state or territory was coded as “unknown” or “refused to give,” and calls where the location was a non-U.S. country or territory (e.g., Mexico, Canada, Micronesia) or an unspecified territory (e.g., overseas U.S. military/diplomatic).

We considered calls received between 2000 and 2021 in U.S. states and territories that reported a single-substance exposure to the NPDS generic codes under the category ichthyosarcotoxins for ciguatera poisoning (CFP), clupeotoxic fish poisoning (CLP), palytoxin poisoning (PLTX), paralytic shellfish poisoning (PSP), scombroid fish poisoning (SFP), tetrodotoxin poisoning (TTX), as well as calls coded as “other types of seafood poisoning” that specified the toxin as azaspiracid shellfish poisoning (AZP), diarrhetic shellfish poisoning (DSP), or domoic acid poisoning (DAP). The NPDS codes tetrodotoxin as “tetrodon poisoning (TTX)”. We use “tetrodotoxin” here instead, as the NPDS code includes exposures to other tetrodotoxin-producing species, not only pufferfish. Total populations for states and territories by sex were downloaded from the U.S. Census Bureau [15]. For territories without intercensal estimates available by sex, estimates were calculated by weighting decennial census counts based on the intercensal year’s decennial census proximity (e.g., 2001 population estimate = 2000 population × 0.9 + 2010 population × 0.1), and the 2021 population was projected using the arithmetic method based on the growth rate observed between the previous two censuses. We used territory-level spatial units for U.S. territories, as this is the smallest spatial unit for which exposure data are available for all territories.

We calculated incidence rate ratios (IRRs) using multilevel mixed-effects negative binomial regression [16]. Call counts were aggregated by year for states and territories and used as the dependent variable; the only exception was for the year 2020, which was treated as two separate periods (i.e., January 2020–April 2020 and May 2020–December 2020) due to the timing of pandemic-related restrictions. Call counts were regressed on a binary independent variable for occurrence during pandemic restrictions, from April 2020 to December 2021 [4]. Population estimates for each state-month were obtained by interpolation. Sex-stratified models followed the same procedures.

To test for Bonferroni significance, we calculated a Bonferroni alpha of ≤0.0009 (i.e., *p* = 0.05/54).

We conducted sensitivity analyses to identify significant trends in the data for further scrutiny (i.e., if a trend observed during the pandemic should be considered a pandemic trend or if the trend began before the pandemic). This analysis was conducted by restricting the pre-pandemic time period to only twice as long as the pandemic period (i.e., January 2016–April 2020).

All statistical analyses were produced using Stata/SE 18.0 (StataCorp, College Station, TX, USA). Figure 1 was produced using the -tsline- function in Stata.

## Figures and Tables

**Table 1:** Incidence rate ratios (IRRs) comparing the rate of calls reporting different marine toxin poisonings in US states and territories over May 2020–December 2021 compared to January 2000–April 2020. IRRs were obtained using multilevel mixed-effects negative binomial regression. * Bonferroni-significant (*p* < 0.0009).

Poisoning		Base	Women	Men
Azaspiracid (AZP)	IRR	0.83	0.88	0.81
	95% CI	(0.46, 1.51)	(0.44, 1.77)	(0.40, 1.65)
	N	328	173	152
Ciguatera (CFP)	IRR	0.57 *	0.58	0.57 *
	95% CI	(0.43, 0.76)	(0.41, 0.81)	(0.41, 0.78)
	N	3058	1458	1540
Clupeotoxic (CLP)	IRR	0.12 *	0.07	0.19
	95% CI	(0.04, 0.39)	(0.01, 0.52)	(0.05, 0.76)
	N	261	147	114
Diarrhetic (DSP)	IRR	0.28 *	0.23 *	0.36
	95% CI	(0.16, 0.49)	(0.10, 0.49)	(0.17, 0.78)
	N	601	331	260
Domoic Acid (DAP)	IRR	0.91	1.01	0.80
	95% CI	(0.47, 1.75)	(0.41, 2.47)	(0.34, 1.90)
	N	209	101	108
Palytoxin (PLTX)	IRR	1.94 *	1.87	2.12 *
	95% CI	(1.32, 2.84)	(0.97, 3.58)	(1.47, 3.07)
	N	644	153	490
Paralytic (PSP)	IRR	0.23 *	0.20 *	0.24 *
	95% CI	(0.17, 0.33)	(0.13, 0.32)	(0.16, 0.37)
	N	5110	2754	2325
Scombroid (SFP)	IRR	0.46 *	0.46 *	0.52 *
	95% CI	(0.36, 0.57)	(0.36, 0.59)	(0.39, 0.68)
	N	3636	1962	1606
Tetrodotoxin (TTX)	IRR	1.73 *	1.73 *	1.83 *
	95% CI	(1.46, 2.04)	(1.38, 2.16)	(1.51, 2.22)
	N	3116	1338	1749

**Table 2:** Incidence rate ratios (IRR) comparing the rate of calls reporting different marine toxin poisonings in US states and territories over May 2020–December 2021 compared to January 2015–April 2020. IRRs were obtained using multilevel mixed-effects negative binomial regression. * Bonferroni-significant (*p* < 0.0009).

Poisoning		Base	Women	Men
Azaspiracid (AZP)	IRR	0.60	0.68	0.52
	95% CI	(0.35, 1.05)	(0.33, 1.39)	(0.27, 0.99)
	N	152	76	75
Ciguatera (CFP)	IRR	0.56 *	0.58	0.57
	95% CI	(0.41, 0.78)	(0.40, 0.85)	(0.40, 0.80)
	N	934	452	469
Clupeotoxic (CLP)	IRR	0.16	0.10	0.22
	95% CI	(0.05, 0.54)	(0.01, 0.83)	(0.05, 0.94)
	N	59	30	29
Diarrhetic (DSP)	IRR	0.52	0.38	0.81
	95% CI	(0.27, 1.00)	(0.17, 0.89)	(0.32, 2.07)
	N	107	63	42
Domoic Acid (DAP)	IRR	1.04	0.91	1.00
	95% CI	(0.52, 2.11)	(0.36, 2.29)	(0.39, 2.53)
	N	74	39	35
Palytoxin (PLTX)	IRR	0.93	0.75	1.05
	95% CI	(0.67, 1.28)	(0.43, 1.31)	(0.76, 1.45)
	N	441	112	328
Paralytic (PSP)	IRR	0.57	0.52	0.62
	95% CI	(0.40, 0.81)	(0.34, 0.79)	(0.41, 0.95)
	N	546	281	263
Scombroid (SFP)	IRR	0.59 *	0.56 *	0.67
	95% CI	(0.44, 0.79)	(0.41, 0.77)	(0.48, 0.93)
	N	917	510	404
Tetrodotoxin (TTX)	IRR	1.71 *	1.69 *	1.86 *
	95% CI	(1.37, 2.15)	(1.29, 2.23)	(1.45, 2.38)
	N	1270	548	710

## Data Availability

America’s Poison Centers owns and maintains the National Poison Data System; data are licensed and reviewed only with careful clinical oversight. Publication of aggregated NPDS data by CDC is permitted through an established data licensing agreement between America’s Poison Centers and CDC.

## Abbreviations

The following abbreviations are used in this manuscript:

PCs	U.S. Poison Centers
NPDS	The National Poison Data System
AZP	Azaspiracid shellfish poisoning
CFP	Ciguatera poisoning
CLP	Clupeotoxic fish poisoning
DSP	Diarrhetic shellfish poisoning
DAP	Domoic acid poisoning
PLTX	Palytoxin poisoning
PSP	Paralytic shellfish poisoning
SFP	Scombroid fish poisoning
TTX	Tetrodotoxin poisoning
IRR	Incidence rate ratio
CI	Confidence interval
